# 

**Published:** 1897-09

**Authors:** 


Vol. XVIII. SEPTEMBER, 1897. No. 9.

PUBLISHED MONTHLY AT $3 A YEAR. SINGLE COPIES, 30 CENTS.

LIBRARY
SURGEON GENERAL'S OFFICE
SEP.-23-1897

### THE JOURNAL OF COMPARATIVE MEDICINE AND VETERINARY ARCHIVES

EDITED AND PUBLISHED BY
RUSH SHIPPEN HUIDEKOPER, Veterinarian (Alfort),
W. HORACE HOSKINS, D.V.S.,
H. D. GILL, V.S.

ALL COMMUNICATIONS AND PAYMENTS SHOULD BE ADDRESSED TO

**Dr. W. Horace Hoskins, Managing Editor, 3452 Ludlow St., Philadelphia, Pa.**

Copies may be had on order of all news-dealers. In London of Baillière, Tindall & Cox.

